# A Tumor-Associated Mutation of FYVE-CENT Prevents Its Interaction with Beclin 1 and Interferes with Cytokinesis

**DOI:** 10.1371/journal.pone.0017086

**Published:** 2011-03-24

**Authors:** Antonia P. Sagona, Ioannis P. Nezis, Kristi G. Bache, Kaisa Haglund, Anne Cathrine Bakken, Rolf I. Skotheim, Harald Stenmark

**Affiliations:** 1 Centre for Cancer Biomedicine, Faculty of Medicine, University of Oslo, Oslo, Norway; 2 Department of Biochemistry, Institute for Cancer Research, The Norwegian Radium Hospital, Oslo University Hospital, Oslo, Norway; 3 Department of Cancer Prevention, Institute for Cancer Research, The Norwegian Radium Hospital, Oslo University Hospital, Oslo, Norway; University of Texas MD Anderson Cancer Center, United States of America

## Abstract

The tumor suppressor activity of Beclin 1 (BECN1), a subunit of class III phosphatidylinositol 3-kinase complex, has been attributed to its regulation of apoptosis and autophagy. Here, we identify FYVE-CENT (ZFYVE26), a phosphatidylinositol 3-phosphate binding protein important for cytokinesis, as a novel interacting protein of Beclin 1. A mutation in FYVE-CENT (R1945Q) associated with breast cancer abolished the interaction between FYVE-CENT and Beclin 1, and reduced the localization of these proteins at the intercellular bridge during cytokinesis. Breast cancer cells containing the FYVE-CENT R1945Q mutation displayed a significant increase in cytokinetic profiles and bi - multinuclear phenotype. Both Beclin 1 and FYVE-CENT were found to be downregulated in advanced breast cancers. These findings suggest a positive feedback loop for recruitment of FYVE-CENT and Beclin 1 to the intercellular bridge during cytokinesis, and reveal a novel potential tumor suppressor mechanism for Beclin 1.

## Introduction

Beclin 1 is a known tumor suppressor protein that regulates apoptosis and autophagy [1]–[3]. Importantly, Beclin 1 is a subunit of the phosphatidylinositol 3-kinase class III (PI3K-III) complex and interacts directly with VPS34, the catalytic subunit of PI3K class III complex [4]–[7]. It also serves as a platform for the recruitment of other proteins such as UVRAG (UV radiation resistance-associated gene) [5], BIF-1/Endophilin B1 [8], and ATG14L/Barkor [9], [10] with known functions in autophagy and tumor suppression. In addition to its known roles in endocytic and autophagic membrane traffic, it was recently established that the PI3K Class III complex plays a crucial role in cytokinesis [11]–[13]. More specifically, the phospholipid PtdIns3*P*, which is produced by VPS34, was found to localize at the intercellular bridge, and depletion of human VPS34 and Beclin 1 resulted in an increased arrest of cells in cytokinesis as well as in an increased amount of binuclear and multinuclear cells [11]. Unsuccessful cytokinesis has been implicated in tumorigenesis but the underlying mechanisms are largely unknown [14].

Here, we uncover a novel potential tumor suppressor mechanism for Beclin 1. We find that Beclin 1 interacts with FYVE-CENT, a PtdIns3*P* binding protein involved in cytokinesis [11]. Further, we show that a tumor-associated mutation of FYVE-CENT abolishes its interaction with Beclin 1, prevents recruitment of Beclin 1 to the intercellular bridge, and is accompanied by cytokinesis arrest and multinuclear phenotype. These results suggest a novel tumor suppressor mechanism for Beclin 1, which is supported by our finding that both Beclin 1 and FYVE-CENT are downregulated in advanced breast cancer.

## Results

### FYVE-CENT is a novel Beclin 1 interacting protein

**Figure 1:**
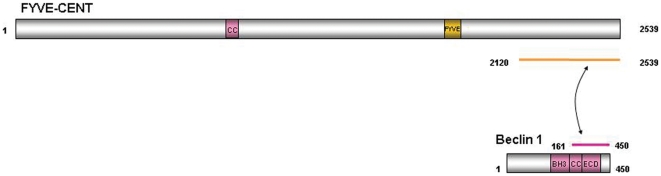
Two-hybrid interactions of Beclin 1 with FYVE-CENT. The figure shows schematically the domain of Beclin 1 that interacts with FYVE-CENT.

**Figure 2:**
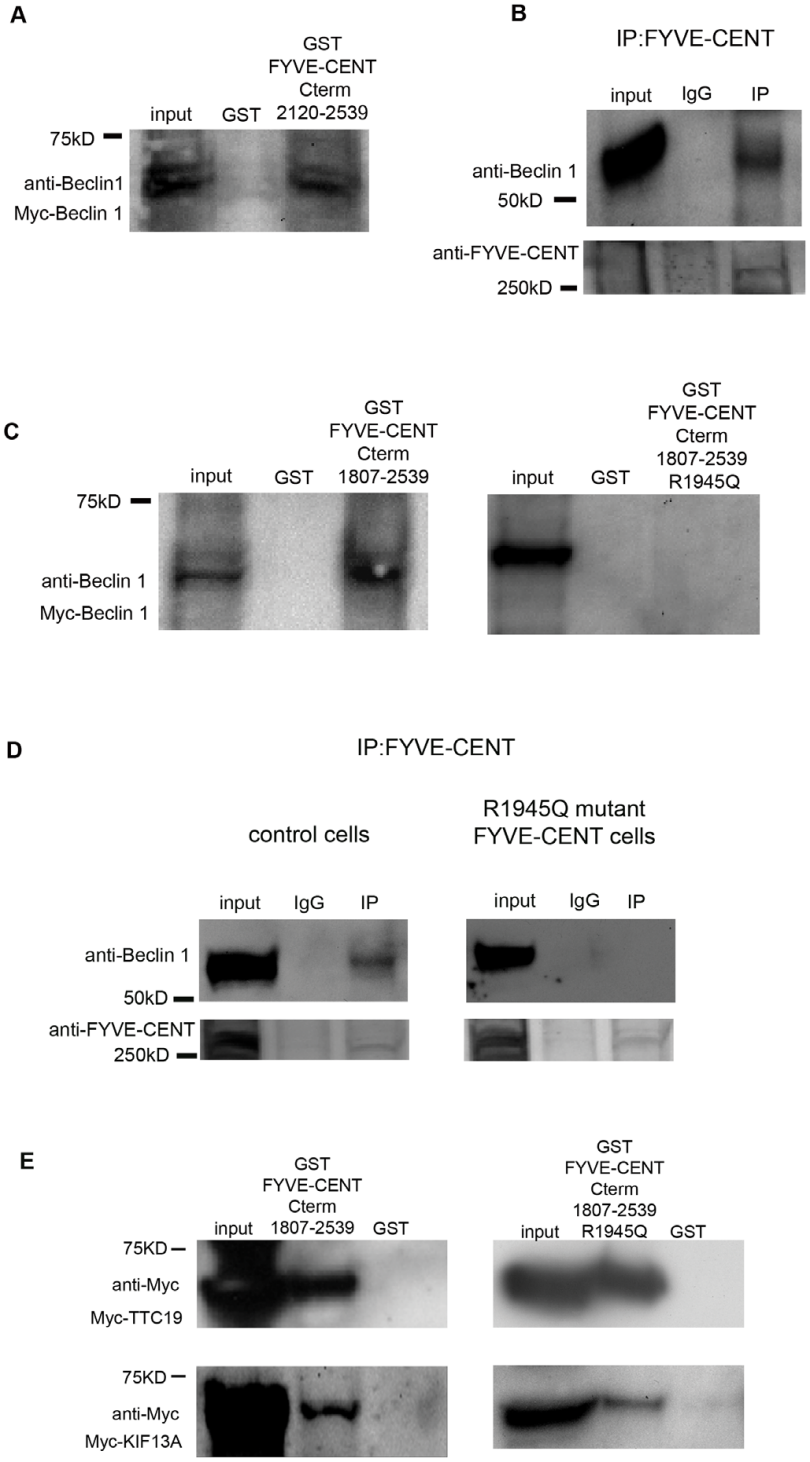
Beclin 1 interacts with FYVE-CENT. (A) GST pull-down from HeLa cell lysates transiently over-expressing myc-Beclin 1 using recombinant GST-FYVE-CENT C-terminal fusion (2120–2539) protein or GST protein immobilized on glutathione-Sepharose beads. Proteins eluted from the beads were analyzed by SDS-PAGE and immuno-blotting using an anti-myc antibody. Equal amounts of GST-FYVE-CENT C-terminal fusion protein and GST protein were loaded. (B) HeLa cell lysates were subjected to immunoprecipitation (IP) with an antibody against FYVE-CENT. Immunoprecipitated proteins were detected by Western blotting, using anti-Beclin 1 and anti-FYVE-CEΝT antibodies. (C) HeLa cells transiently over-expressing myc-Beclin 1 were pulled down with recombinant GST-FYVE-CENT C-terminal fusion (1807–2539 or 1807–2539 R1945Q) protein or GST protein immobilized on glutathione-Sepharose beads. (D) HCC-1395 control cells and HCC-1954 FYVE-CENT R1945Q mutant cells were lysed and subjected to immunoprecipitation (IP) with an antibody against FYVE-CENT. Immunoprecipitated proteins were detected by Western blotting, using anti-Beclin 1 and anti-FYVE-CEΝT antibodies. (E) HeLa cells transiently over-expressing myc-TTC19 or myc-KIF13A were pulled down with recombinant GST-FYVE-CENT C-terminal fusion protein and GST-FYVE-CENT C-terminal R1945Q fusion protein or GST protein immobilized on glutathione-Sepharose beads.

We have recently shown that FYVE-CENT is a critical PtdIns3*P* effector protein that regulates cytokinesis [11]. In order to identify interacting partners of FYVE-CENT, we performed a yeast two-hybrid screen in a human T-lymphocyte library, using the C-terminal part of FYVE-CENT as bait (residues 2120–2539). Using this approach, Beclin 1 was identified as a positive hit (Dataset S1). The interaction of Beclin 1 with FYVE-CENT maps to a region containing the coiled coil domain and the evolutionarily conserved domain of Beclin 1 (Figure 1). To verify this interaction biochemically, we performed a pull-down assay, incubating the C-terminus of FYVE-CENT fused to GST with myc-Beclin 1 expressed in HeLa cell lysates. The pull-down assay showed a positive biochemical interaction (Figure 2A). To further verify this interaction, endogenous FYVE-CENT and Beclin 1 were co-immuno-precipitated with an antibody against FYVE-CENT (Figure 2B), indicating that the two endogenous proteins can form a complex in vivo.

### A mutation associated with breast cancer abolishes the interaction between FYVE-CENT and Beclin 1

The *ZFYVE26* gene encoding FYVE-CENT was found mutated in breast cancer samples with a frequency of more than 10% [15]. Since Beclin 1 is a well-known tumor suppressor [2], [16], we therefore wanted to test the cell biological consequence of such mutations in the context of FYVE-CENT interaction. To this end we performed a GST pull-down between the C-terminal part of FYVE-CENT (residues 1807–2539) that contains the R1945Q mutation found in breast cancer cell lines [15] and myc-Beclin 1 in HeLa cell lysates. Interestingly we observed that the FYVE-CENT R1945Q mutation abolished the interaction between FYVE-CENT and Beclin 1 (Figure 2C). This was also confirmed in co-immuno-precipitation experiments where endogenous FYVE-CENT R1945Q mutant protein extracted from HCC-1954 breast cancer cells and Beclin 1 did not co-immuno-precipitate with an antibody against endogenous FYVE-CENT, whereas wild-type FYVE-CENT from a control cancer cell line was able to co-immuno-precipitate with Beclin 1 (Figure 2D). We have previously shown that FYVE-CENT interacts with the microtubule-based motor KIF13A and the tetratricopeptide repeat protein TTC19 [11]. KIF13A was found to regulate translocation of FYVE-CENT to the midbody, and the importance of these proteins in cytokinesis is illustrated by the finding that depletion of either FYVE-CENT, KIF13A or TTC19 is sufficient to cause an increased number of cytokinetic profiles and bi- and multinucleate cells [11]. We therefore asked whether the FYVE-CENT R1945Q mutation also interferes with its interaction with KIF13A and TTC19. Interestingly, pull-down assays showed that the R1945Q mutation does not inhibit the interaction of the C-terminus of FYVE-CENT with neither TTC19 nor KIF13A in vitro (Figure 2E). These data indicate that the FYVE-CENT R1945Q mutation associated with breast cancer specifically abolishes the interaction of FYVE-CENT with Beclin 1.

### Breast cancer cells containing the FYVE-CENT R1945Q mutation display a significant increase in cytokinetic profiles and hyperploidy

**Figure 3:**
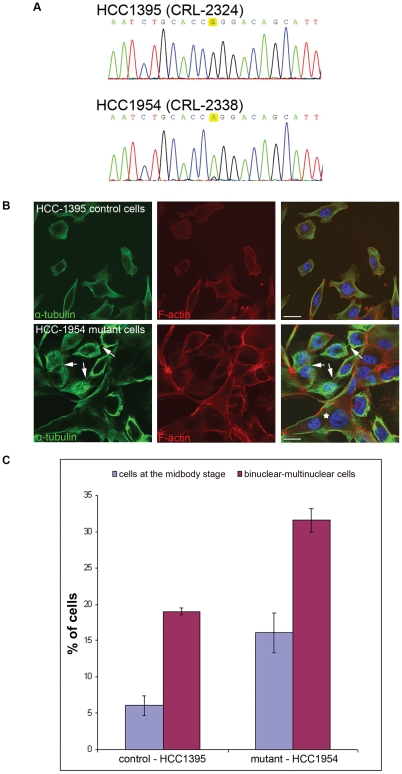
A FYVE-CENT R1945Q mutant breast cancer cell line exhibits an increased number of cells arrested in cytokinesis as well as bi- and multinuclear cells. (A) Sequencing of cDNA for exons 31 to 33 of *FYVE-CENT* from the HCC-1395 and HCC1954 breast cancer cell lines revealed a G to A substitution at base position 5834 in the HCC1954 cell line. (B). Confocal micrographs of HCC-1395 and HCC-1954 breast cancer cell lines cells stained with α-tubulin, Alexa Fluor® 594 phalloidin and Hoechst. In FYVE-CENT mutant cells (HCC-1954) there is a significant increase in cells arrested in cytokinesis (arrows) compared to the control as well as increase in binuclear-multinuclear cells (asterisk). Scale bars: 20 µm. (**C**) Graphic presentation of quantification of cells arrested at the midbody stage and bi-multinuclear cells in control cells (HCC-1395) and FYVE-CENT R1945Q mutant cell line (HCC-1954). Error bars show mean ± s.d. Control: 3 independent experiments, n = 1142 cells. Mutant cells: 3 independent experiments, n = 1225 cells. p value for cells arrested at the midbody stage <0.01. p value for binuclear-multinuclear cells <0.01.

In order to examine the biochemical consequences of the cancer-associated R1945Q mutation of FYVE-CENT, we investigated the HCC-1954 breast cancer cell line which contains this mutation [15]. By cDNA sequencing, we confirmed the mutation status of the cell line and also that the mutant gene is indeed expressed (Figure 3A). Interestingly, cDNA sequencing detected almost exclusively the mutant allele and only a weak signal for the wild-type, indicating a preferential expression of the mutant allele in a heterozygous cell line, or alternatively, that only the mutant allele is present in the majority of the cells, and the existence of a sub-population of cells which is heterozygous for the mutation. The protein levels of Beclin 1 were comparable in the cell line used as control (HCC-1395) and the mutant FYVE-CENT (HCC-1954) cells (Figure S1A). Consistent with this, upon siRNA depletion of FYVE-CENT, Beclin 1 protein levels remained the same (Figure S1B). Likewise, FYVE-CENT levels ramained unaffected by depletion of Beclin 1. In contrast, upon depletion of the Beclin 1 interacting protein VPS34, Beclin 1 became downregulated whereas FYVE-CENT protein levels remained the same (Figure S1B). These results show that the FYVE-CENT R1945Q mutation does not affect the protein levels of Beclin 1.

In order to identify any biological consequence of the FYVE-CENT R1945Q mutation, we examined the phenotype of mutant cells by performing immunofluorescence microscopy using the HCC-1954 and HCC-1395 breast cancer cells. We observed that FYVE-CENT R1945Q mutant cells showed an increased population arrested in cytokinesis (16%) compared to the control cells (6%) and also an increased percentage of binuclear-multinuclear profiles (31.5% versus 19%) (Figure 3B–C and Figure S2A–C). In order to examine whether this phenotype is a direct consequence of the FYVE-CENT R1945Q mutation, we tested whether R1945Q mutation can rescue the arrest in cytokinesis observed upon FYVE-CENT depletion. To examine this we back-transfected HeLa cells which were RNAi-depleted for FYVE-CENT with wild type FYVE-CENT C terminus (1807–2539) or FYVE-CENT C terminus R1945Q mutant. We observed that wild type FYVE-CENT C-terminus could rescue the arrest in cytokinesis and bi-multinuclear phenotype observed upon FYVE-CENT RNAi depletion suggesting that this part of FYVE-CENT entails the minimal functional domains. In contrast, the FYVE-CENT C terminus R1945Q mutant could not rescue the siRNA-induced phenotypes (Figure S3A–C). These data suggest that the FYVE-CENT R1945Q mutation may promote carcinogenesis by interferring with normal cytokinesis.

### Beclin 1 localizes at the intercellular bridge during cytokinesis, and this localization is abolished in FYVE-CENT R1945Q mutant breast cancer cells

**Figure 4:**
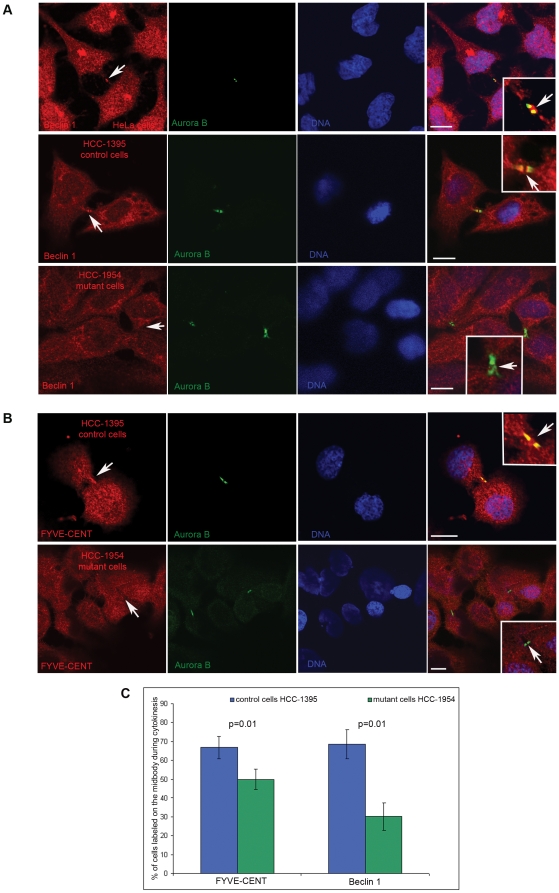
The localization of Beclin 1 to the intercellular bridge during cytokinesis is abolished in FYVE-CENT R1945Q mutant breast cancer cells. (A) and (B) Confocal micrographs of HeLa, HCC-1395 and HCC-1954 cells stained with antibodies against Aurora B and Beclin 1 (A) or FYVE-CENT (B), and with Hoechst. Magnifications of the intercellular bridges are shown in the insets. Scale bars: 10 µm. (C) Graphic presentation of quantification of control cells (HCC-1395) and mutant cells (HCC-1954) labeled on the midbody with anti-FYVE-CENT or anti-Beclin 1 antibodies. Error bars show mean ± s.d. Control cells stained with anti-FYVE-CENT: 4 independent experiments, n = 1769 cells. Mutant cells stained with anti-FYVE-CENT: 4 independent experiments, n = 1781 cells. Control cells stained with anti-Beclin 1: 4 independent experiments, n = 1340. Mutant cells stained with anti-Beclin 1: 4 independent experiments, n = 1521. p value for cells labeled with anti-FYVE-CENT on the midbody: 0.01. p value for cells labeled with anti-Beclin 1 on the midbody: 0.01.

We next asked how the interplay between FYVE-CENT and Beclin 1 may regulate cytokinesis. We have recently shown that VPS34, the catalytic subunit of PI3K-III complex, and FYVE-CENT are localized at the intercellular bridge during cytokinesis [11]. Given the interaction of Beclin 1 with FYVE-CENT, we examined the localization of Beclin-1 during cytokinesis, and we found that this protein also localizes at the intercellular bridge (Figure 4A, upper panels). Interestingly, Beclin 1 was also found to localize at the intercellular bridge in the control cell line HCC-1395, whereas in the FYVE-CENT R1945Q mutant breast cancer cell line this localization was significantly reduced (Figure 4A and 4C). Additionally, the localization of FYVE-CENT at the intercellular bridge was partially abolished in the FYVE-CENT R1945Q mutant breast cancer cell line (Figure. 4B and 4C). Taken together, these data suggest that the FYVE-CENT R1945Q mutation prevents localization of Beclin 1 at the intercellular bridge and interferes with proper cytokinesis.

### Downregulation of FYVE-CENT and Beclin 1 in advanced breast cancer

**Figure 5:**
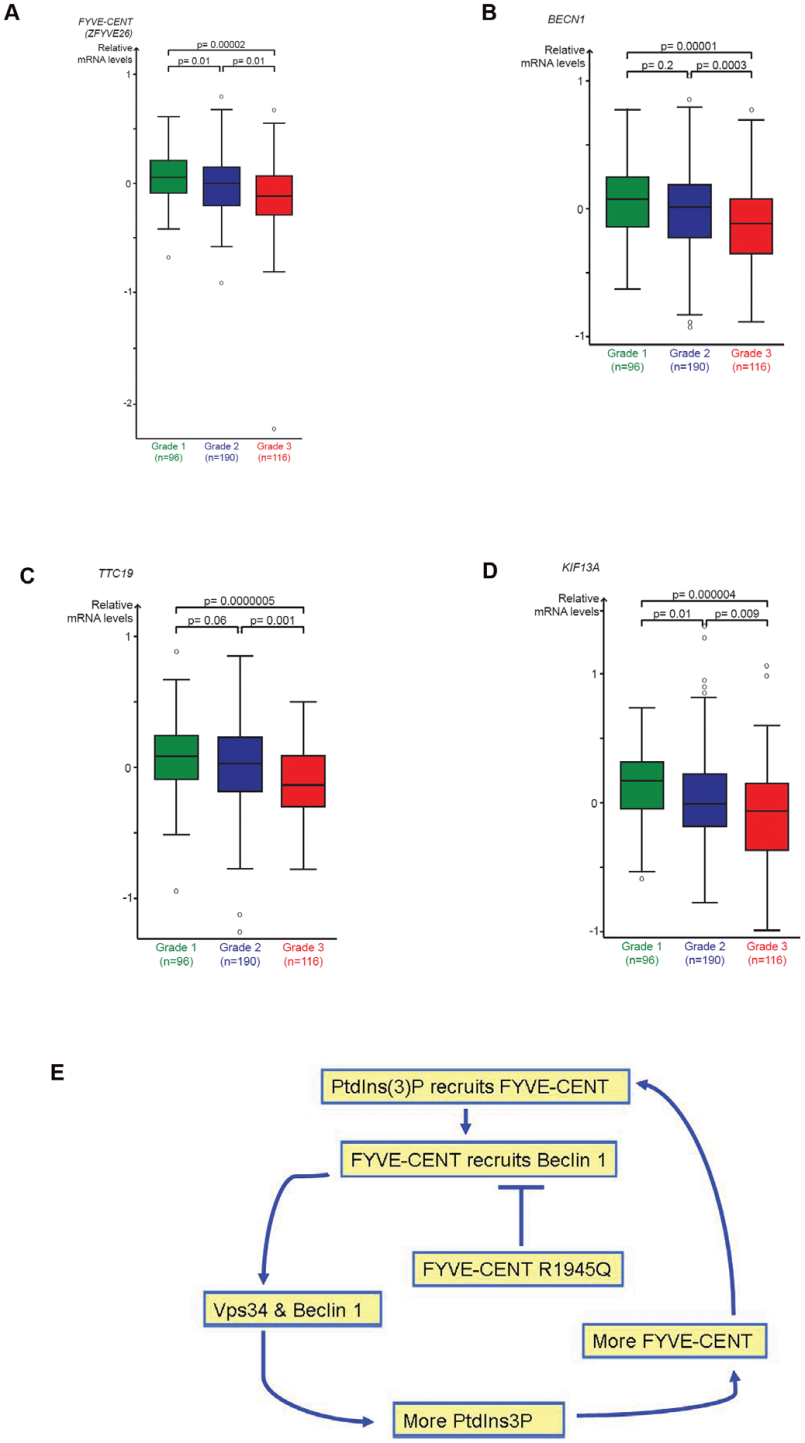
The average expression of *FYVE-CENT* and associated genes is significantly lower in high vs. low grade breast cancers. (A)–(D) The gene expression data were derived from the Gene Expression Omnibus GSE1456 and GSE4922 datasets. The expression values were median centred within each of the series separately. The p-values were derived from comparing means by independent samples t-test (SPSS, v.16.0). (E) Proposed model: FYVE-CENT- Beclin 1 interplay in a positive feedback loop manner during cytokinesis.

To further explore the association of FYVE-CENT with breast cancer, we examined its expression pattern in previously published gene expression data [17], [18]. We found that the average expression of *FYVE-CENT* was significantly lower in high vs. low grade breast cancers (Figure 5A). Furthermore, we found that there was a similar significant association between decreased *BECN1* mRNA levels and tumor grade (Figure 5B). More specifically, breast cancers of grade 3 had a significantly lower expression mean than grade 1 and 2 tumors. Interestingly, we also observed that the average expression of *KIF13A* and *TTC19* was significantly lower in high vs. low grade breast cancers (Figure 5C and 5D). Altogether, the associations to clinical parameters strengthen the links between FYVE-CENT, Beclin 1 and breast cancer biology.

## Discussion

The tumor suppressor activity of Beclin 1 has been attributed to its interactions with proteins that regulate cell death and autophagy [2], [3], [7]. Our present data suggest an additional mechanism for the tumor suppressor functions of Beclin 1, namely its ability to bind FYVE-CENT and participate in the regulation of cytokinesis. Failure to complete cytokinesis has been implicated in carcinogenesis [14], [19], [20], and our data demonstrate that the Beclin 1 - FYVE-CENT complex may play important roles in controlling this process. Importantly, mutations in FYVE-CENT associated with breast cancer interfere with its interaction with Beclin 1. It is interesting that loss of this interaction is accompanied by cytokinesis failure, since this suggests a mechanism that may contribute to the cancer phenotype of FYVE-CENT mutant cancer cells.

The fact that the R1945Q mutation is located outside the minimal interacting part of FYVE-CENT with Beclin 1 may suggest that there are additional interacting surfaces that extend outside the 2120–2539 C-terminal part that was used as bait in the yeast two- hybrid screen. Alternatively, the R1945Q mutation might promote a conformational change in C-terminal folding that could alter its association with Beclin 1, or result in recruitment of chaperone proteins that would sterically prevent Beclin 1 binding. The R1945Q mutation does not affect the interaction of FYVE-CENT with KIF13A and TTC19, suggesting that it specifically abolishes binding to Beclin-1. The downregulation of *FYVE-CENT*, *BECN 1*, *KIF13A* and *TTC19* in advanced breast cancer is consistent with the possibility that these proteins may participate in tumor suppression.

We have recently shown that PtdIns3*P* recruits FYVE-CENT at the midbody during cytokinesis, and that subunits of the PI3K-III complex, including Beclin 1, are required for correct cytokinesis [11]. Our present data suggest a positive-feedback loop model wherein FYVE-CENT can recruit Beclin 1 at the intercellular bridge. Subsequently, Beclin 1 can interact with VPS34, thereby producing more PtdIns3*P*, which in turn can recruit more FYVE-CENT. This model (Figure 5E) would explain the significant increase in cells arrested in cytokinesis and bi- and multinuclear cells in FYVE-CENT mutant cells and highlight a role for Beclin 1 in cytokinesis. Collectively, our findings reveal a novel regulatory role of the tumor suppressor Beclin 1 and its binding partner FYVE-CENT that has potential implications for carcinogenesis.

## Materials and Methods

### Cell culture and transfections

HeLa cells were grown and transfected as described previously [11]. HCC-1395 (CRL-2324) and HCC-1954 (CRL-2338) cells were purchased from ATCC and grown in RPMI-1640 medium (GIBCO, Invitrogen) supplemented with 10% fetal bovine serum in a 5% CO_2_ atmosphere at 37°C.

### Confocal fluorescence microscopy

Immunofluorescence microscopy was performed using HeLa, HCC-1395 and HCC-1954 as previously described [11]. The following primary antibodies were used for immunofluorescence studies: rabbit anti-human FYVE-CENT antibody, used in 1∶300 dilution, as described before [11], mouse anti-α-tubulin, used in 1∶1000 dilution and purchased from SIGMA, rabbit anti-human Beclin 1 and mouse anti-human Aurora B antibody, both used in 1∶200 dilution and purchased from Abcam. The secondary antibodies used were goat-anti-mouse Alexa Fluor® 488, in 1∶500 dilution from Invitrogen and Cy3-labelled goat anti-rabbit antibody, in 1∶500 dilution and Cy2-labelled goat anti-mouse antibody, in 1∶200 dilution purchased from Jackson Immunoresearch. Alexa Fluor® 594 phalloidin, used in 1∶750 dilution, and Hoechst 33342, used at 1 µg/µl, were purchased from Invitrogen.

### Immunoblotting

To determine the cell-specific distribution of FYVE-CENT, Beclin 1, VPS34, beta-actin and the overexpressed TTC19 and KIF13A-myc tagged constructs, the various cell lines were lysed in lysis buffer (25 mM HEPES pH 7.2, 125 mM potassium acetate, 2.5 mM magnesium acetate, 5 mM EGTA, 1 mM DTT, 0.5% Nonidet P40, 1∶100 proteinase inhibitor mix (Roche Applied Science). After centrifugation for 5 min at 5,000 *g* the samples were sonicated for 10 s at 70 volts and incubated for 10 min on ice in lysis buffer. Another centrifugation at 10,000 g separated the supernatant from the pellet and 30 µg of protein of the supernatant was subjected to SDS–PAGE (4–20% gradient) and transferred to Immobilon-P membrane (Millipore) for immunoblotting. The blot was developed with the Supersignal West Pico Chemiluminescent substrate kit or Supersignal West Femto Maximum Sensitivity Substrate kit (Pierce). The antibodies used for immunoblotting were the following: Rabbit anti-human Beclin 1 antibody used for western blotting and immunoprecipitation, was purchased from Cell Signaling Technology. Rabbit c-Myc polyclonal antibody was purchased from Abcam and the rest antibodies used (anti-FYVE-CENT, anti-VPS34, anti-beta-actin, anti-GST and HRP labeled) were described previously [11]. For quantitative Western blotting, equal amounts of cell lysates (as measured by protein content) from control and mutant cells were loaded in triplicates on a gel for PAGE. The proteins were transferred to a PVDF membrane and stained with antibodies for FYVE-CENT, Beclin1 and β-actin. The bands were detected using LiCore infrared dye secondary antibodies and the Odyssey imaging system. The bands were quantified using the Odyssey quantifying software.

### GST pull-down assay

The GST–FYVE-CENT C-terminus ( amino acid residues 2120–2539), the GST–FYVE-CENT C-terminus (1807–2539) and the GST–FYVE-CENT C-terminus (1807–2539) mutant R1945Q constructs were expressed in BL21 *Escherichia coli*, purified and GST-pull down assays were performed as described previously [11].

### Co-immunoprecipitation analysis

Rabbit antibody against FYVE-CENT or rabbit IgG (control) were rotated at RT (room temperature) with Protein A agarose beads for 1 h. Then the beads were washed two times with PBS and two times with 0.2 M triethanolamine, pH 8.2. Crosslinking was performed by rotating the beads in 0.2 M triethanolamine containing 3 mg/ml dimethyl pimelimidate at 4°C overnight. In order to quench the unreacted beads, they were rotated with 10 mM ethanolamine, pH 8.2, at 4°C for 30 min. The beads were washed three times with PBS and were used for immunoprecipitation.

HeLa, HCC-1395 and HCC-1954 cells were grown confluent in 10-cm culture dishes and lysed in ice-cold lysis buffer (20 mM HEPES pH 7.2, 2 mM MgCl2, 100 mM NaCl, 0.1 mM EDTA, 0.1% Triton X-100) containing inhibitors (*N*-ethylmaleimide, mammalian protease inhibitor mixture, phosphatase inhibitor cocktail I and II (Sigma-Aldrich).The lysates were placed on ice and centrifuged at 10,000 g, 4°C and the supernatant was added to the Protein A-coupled magnetic beads (Dynal, Invitrogen) which had been precoupled with rabbit antibody against FYVE-CENT or rabbit IgG as a control, in PBS Tween 20. Antibody coupled magnetic beads and cell lysates were gently mixed for 1 h at 4°C. The beads were then washed with lysis buffer, eluted in 4× sample buffer plus 1 mM DTT at 95°C for 5 min. The eluted proteins were subsequently subjected to SDS–PAGE and immunoblotting as described previously.

### Plasmid constructs

All the FYVE-CENT constructs used were generated by PCR with the *FYVE-CENT* cDNA (ORF) (NM_015346.2), which was cloned in a pCMV6-XL4 vector by OriGene Technologies, Inc., as template. Synthetic oligonucleotides were from MWG Biotech. The FYVE-CENT R1945Q mutant was prepared by PCR site-directed mutagenesis. PCR errors were excluded by sequencing. For expression as GST fusion proteins in *Escherichia coli* BL21 (DE3) cells, the C-terminal part (2120–2539) as well as (1807–2539) and with mutation (R1945Q) of FYVE-CENT were cloned into pGEX-6P-3 (Pharmacia Amersham). The expression plasmid encoding myc-epitope-tagged mouse KIF13A and the Myc-DDK-tagged ORF clone of Homo sapiens TTC19 (NM_017775.2) were obtained as described previously [11]. Expression in mammalian cells and purification were performed as described previously [11].

### Assay of rescuing cytokinesis phenotype in RNAi FYVE-CENT depleted cells

HeLa cells were transfected with siRNA (70 nM) against human FYVE-CENT for 72 h. The siRNA-treated cells were then seeded onto coverslips in a 5 cm culture dish and were transfected with myc-tagged C- terminal 1807–2539 and myc-tagged C-terminal 1807–2539 R1945Q FYVE-CENT constructs respectively in three different series of experiments for 36 h. The cells were washed in PBS, stained with anti-myc and anti-α tubulin antibodies and processed in confocal microscopy analysis as described above. The experiment was repeated three times and in total, and 270 back transfected cells were quantified. In parallel, simple depletion experiments using control and FYVE-CENT siRNA were performed in triplicates and quantified using the same stainings and conditions.

### RNA interference studies

Single deconvoluted siRNAs against FYVE-CENT (cat.no. D-031136-04), VPS34 (PIK3C3)(cat. no. D-005250-04) and Beclin 1(siRNA 1: cat. no. J-010552-05) were purchased from Dharmacon Research. The siRNA experiments were performed on HeLa cells as described before [11].

### Yeast two-hybrid screening

The yeast two-hybrid screening was based on the C terminus (residues 2120–2539) of FYVE-CENT as bait and performed by Hybrigenics S.A Services using a human T cells RP1 (CEMC7) library.

### RNA isolation/cDNA sequencing

Total RNA was isolated from HCC-1395 (control cells) and HCC-1954 (FYVE-CENT R1945Q mutants cells) (1 well of a 6 well plate for each) using the Total RNA Mini Kit (BioRad) according to the manufacturer's descriptions. One microgram RNA was converted to cDNA using the iScript cDNA Synthesis kit (BioRad). Forward and reverse primers, AGGAGGAAAATGAGCTGGTG and CAGCACATCTACCTTGCTGA, were designed with the Primer3 software using default settings, and PCR products were sequenced in forward and reverse using an ABI 3730 DNA Analyzer (Life Technologies).

### Statistical analysis

Values are given as means and s.d in all figures. The p values are calculated based on t-test.

## Supporting Information

Figure S1 **FYVE-CENT and Beclin 1 expression in HCC-1395 and HCC-1954 breast cancer cells.** (A) Whole cell lysates from HCC-1395 and HCC-1954 (FYVE-CENT R1945Q mutant) cell lines were analyzed by immunoblotting with the indicated antibodies. Equal amounts of cell lysates (as measured by protein content) from control and mutant cells were loaded in triplicates. The bands were detected using LiCore infrared dye secondary antibodies and the Odyssey imaging system. The bands were quantified using the Odyssey quantifying software, and the numbers resulting from the average of three loadings are shown. (B) Whole cell lysates from HeLa cells transfected with scrambled (scr) or the indicated siRNAs were analyzed by immunoblotting with the indicated antibodies. Experiments were repeated three times and a representative blot is shown.
(TIF)

Figure S2 **A FYVE-CENT R1945Q mutant breast cancer cell line exhibits an increased number of cells arrested in cytokinesis as well as bi- and multinuclear cells.** (A–B) Confocal micrographs of HCC-1395 and HCC-1954 breast cancer cells stained with the Aurora B and Hoechst (A), and HCC-1954 breast cancer cells stained with the α-tubulin, Alexa Fluor® 594 phalloidin and Hoechst (B). In FYVE-CENT mutant cells (HCC-1954) there is a significant increase in cells arrested in cytokinesis (arrows) compared to the control (A) as well as increase in binuclear-multinuclear cells (B). Scale bars: 10 µm. (C) Graphic presentation of quantification of cells arrested at the midbody stage and bi-multinuclear cells in FYVE-CENT control (HCC-1395) and R1945Q mutant cell lines (HCC-1954). Error bars show mean ± s.d. Control: 6 independent experiments, n = 1982 cells. Mutant cells: 6 independent experiments, n = 2001 cells. p value for cells arrested at the midbody stage: 0.001. p value for binuclear-multinuclear cells: 7×10^−7^.
(TIF)

Figure S3 **Back-transfection of R1945Q FYVE-CENT mutant C terminus (1807–2539) transgene does not rescue cytokinesis arrest caused by siRNA compared to FYVE-CENT C terminus (1807–2539).** Hela cells were tranfected with myc- FYVE-CENT C terminus (1807–2539) and siRNA against FYVE-CENT (A), or myc- FYVE-CENT C terminus (1807–2539) R1945Q mutant and siRNA against FYVE-CENT (B) transgenes simultaneously. The cells expressing myc- FYVE-CENT C terminus (1807–2539) transgene can rescue arrest in cytokinesis compared to the adjacent cells (A) but myc- FYVE-CENT C terminus (1807–2539) R1945Q cannot (B) (arrows). (C), Quantification of the results shown in (A) and (B). Scale bars 10 µm.
(TIF)

Dataset S1 **Positive hits from yeast two-hybrid screening with the C-terminus of FYVE-CENT.** A list of the interacting proteins with the C-terminus (residues 2120–2539) of ZFYVE26 (FYVE-CENT) were identified in a two-hybrid screen of a human T cells RP1 (CEMC7) cell library. The data are from Hybrigenics S.A, Paris, France.
(XLS)
